# Evaluation of the *in Vitro* Anti-Atherogenic Properties of Lipid Fractions of Olive Pomace, Olive Pomace Enriched Fish Feed and Gilthead Sea Bream (*Sparus aurata*) Fed with Olive Pomace Enriched Fish Feed

**DOI:** 10.3390/md11103676

**Published:** 2013-09-30

**Authors:** Constantina Nasopoulou, Vassiliki Gogaki, Giorgos Stamatakis, Leonidas Papaharisis, Constantinos A. Demopoulos, Ioannis Zabetakis

**Affiliations:** 1 Laboratory of Food Chemistry, Faculty of Chemistry, School of Sciences, National and Kapodistrian University of Athens, Athens 15771, Greece; E-Mails: vassilikigogaki@hotmail.com (V.G.); izabet@chem.uoa.gr (I.Z.); 2 Laboratory of Biochemistry, Faculty of Chemistry, School of Sciences, National and Kapodistrian University of Athens, Athens 15771, Greece; E-Mails: stamatakisgeo@gmail.com (G.S.); demopoulos@chem.uoa.gr (C.A.D.); 3 Research and Development Department, Nireus Aquaculture SA, Koropi 19400, Greece; E-Mail: l.papaharisis@nireus.com

**Keywords:** gilthead sea bream (*Sparus aurata*), olive pomace, HPLC, platelet activating factor, anti-atherogenic properties

## Abstract

Given the pivotal role of Platelet-Activating-Factor (PAF) in atherosclerosis and the cardio-protective role of PAF-inhibitors derived from olive pomace, the inclusion of olive pomace in fish feed has been studied for gilthead sea bream (*Sparus aurata*). The aim of the current research was to elucidate the anti-atherogenic properties of specific HPLC lipid fractions obtained from olive pomace, olive pomace enriched fish feed and fish fed with the olive pomace enriched fish feed, by evaluating their *in vitro* biological activity against washed rabbit platelets. This *in vitro* study underlines that olive pomace inclusion in fish feed improves the nutritional value of both fish feed and fish possibly by enriching the marine lipid profile of gilthead sea bream (*Sparus aurata*) with specific bioactive lipid compounds of plant origin.

## 1. Introduction

Fish and fish oil (FO) can significantly reduce the risk of cardiovascular diseases (CVDs) [1,2,3] and there is evidence that FO has the ability to decelerate the formation of plaque in the arteries [4]. Therefore, fish consumption is on the rise, not only due to the increased demand from a growing global population, but also to the widening knowledge of the health benefits derived from its consumption.

On the other hand, one of the biggest issues that aquaculture industry has to face, is the high dependence on FO used for aquaculture fish feed production, combined with the fact that FO and fish meals would reach their limit of sustainability in the next few years. This situation creates an emerging necessity for usage of alternative dietary lipid sources in formulated fish feed, such as plant-derived oils. Research on alternative dietary lipid sources of plant origin has grown considerably over the past few years [5]. Such a promising plant lipid source is olive pomace (OP), which is a by-product of olive oil industry with important cardioprotective properties [6].

Because of this potential use of OP in formulated aquaculture fish feeds, our group has studied the production of aquacultured gilthead sea bream (*Sparus aurata*) using OP diet [7,8]. According to these studies, OP inclusion in aquaculture fish feed did not moderate fish growth performance [7], the nutritional value of fish fillets was improved [7,8], and the sensory perception of the fish was satisfactory [8].

The purpose of the current research was to further elucidate the anti-atherogenic (*i.e.*, cardioprotective) properties of specific HPLC polar lipid fractions of OP, both diets (FO and OP diet) and both fish fillets (fish fed with FO and OP diet) by evaluating their *in vitro* biological activity against washed rabbit platelets [9] and, therefore, to assess the nutritional value of OP diet and of fish fed with OP diet.

## 2. Results

### 2.1. Fish Diets’ Analysis

The OP diet was analyzed for a number of nutritional parameters and results are given in Table 1. To enable easy comparison, the corresponding data of the FO diet (previously published by our team, [7]) are also given in Table 1.

**Table 1:** Chemical composition of olive pomace (OP) and fish oil (FO) diet (% wet weight).

Ingredient	OP diet	FO diet *
Crude protein	44.95 ± 1.3	46 ± 4.3
Fat	19.4 ± 1.7	21 ± 2.1
Moisture	8.6 ± 0.6	9.1 ± 1.3
Dietary fiber	5.2 ± 0.3 ^†^	1.8 ± 0.3 ^†^
Ash	6.0 ± 0.9	8.3 ± 1.4
Energy (MJ/kg)	21.8 ± 2.1	23 ± 2.6
Protein digestibility (%)	89 ± 4.4	90 ± 6.2
Vitamin Α (IU/kg)	7000 ± 210 ^†^	20,000 ± 410 ^†^
Vitamin D (IU/kg)	3150 ± 110	3000 ± 120
Vitamin E (mg/kg)	180 ±17 ^†^	258 ± 19 ^†^
Vitamin K3 (mg/kg)	10 ± 0.7 ^†^	33 ± 7.3 ^†^
Vitamin C (mg/kg)	200 ± 20	168 ± 14
Cu (mg/kg)	7.5 ± 1.1	7.0 ± 1.1

Values are means of three individual measurements; results are expressed as mean ± SD (95% confidence limits); * Data of FO diet are from our previous work [7] and are given here to enable easy comparison; ^†^ Indicates statistical significance within OP and FO diet, according to Wilcoxon test.

### 2.2. Total Lipid (TL), Total Neutral Lipid (TNL), and Total Polar Lipid (TPL) Content of Samples

TL, TPL, and TNL content of OP, FO diet, OP diet, and gilthead sea bream fillets fed with FO and OP diet is shown in Table 2.

**Table 2:** Total lipid (TL), total neutral lipid (TNL), and total polar lipid (TPL) content (%) of olive pomace (OP), fish oil (FO) diet, olive pomace (OP) diet, and fillets of aquacultured gilthead sea bream (*Sparus aurata*) fed with FO and OP diet.

	TL (%)	TNL (%)	TPL (%)
OP	2.4 ± 0.2	0.2 ± 0.02	2.2 ± 0.2
FO diet	21 ± 2.1	11 ± 1.5	10 ± 0.9
OP diet	19 ± 1.3	8.4 ± 1.1	11 ± 0.9
Fish fed with FO diet	3.9 ± 0.3 ^†^	3.7 ± 0.3 ^†^	0.2 ± 0.03 ^†^
Fish fed with OP diet	5.4 ± 0.5 ^†^	4.9 ± 0.4 ^†^	0.5 ± 0.06 ^†^

Values are means of three individual measurements; results are expressed as mean ± SD (95% confidence limits); ^†^ Indicates statistical significance within fish fed with FO and OP diet, according to Wilcoxon test.

According to Table 2, fish fed with OP diet exhibited significant elevated levels of TL in comparison to the ones of fish fed with FO diet. This upward trend is also reflected in TNL and TPL levels of fish fed with OP diet.

### 2.3. HPLC TPL Purification—Biological Assay

**Figure 1:**
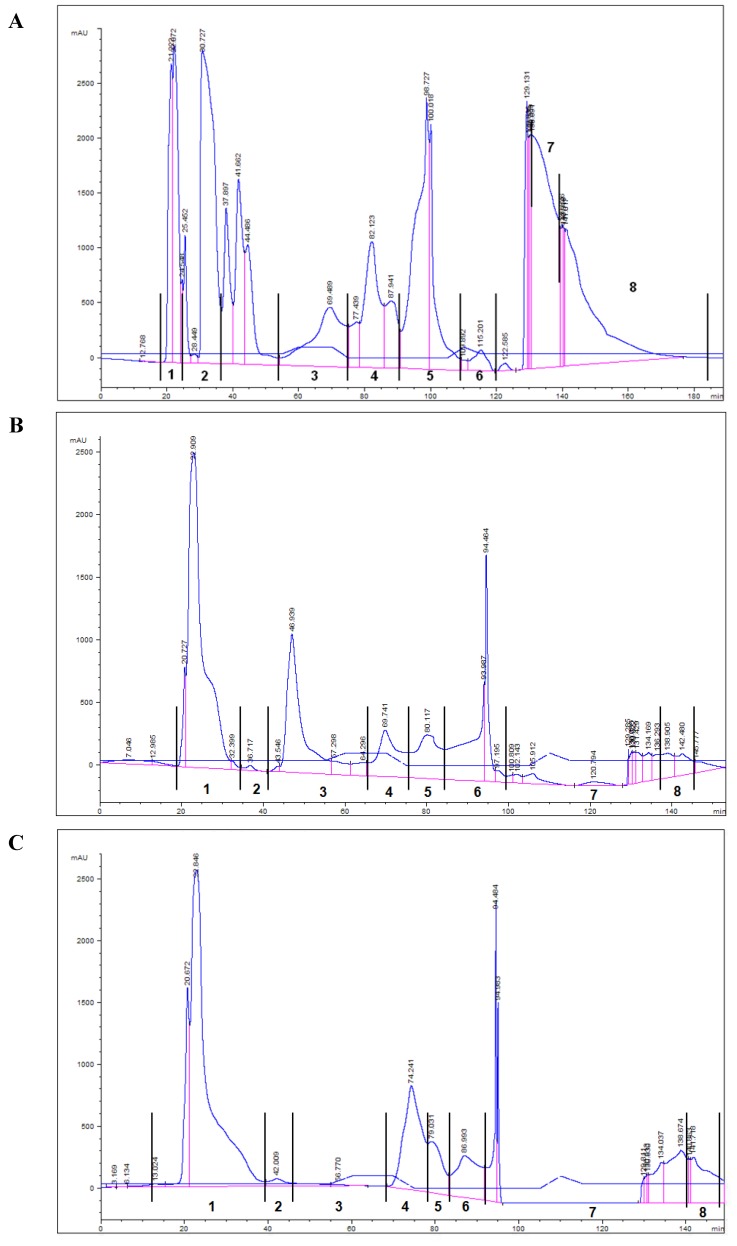
Representative chromatographs by normal-phase HPLC separation of total polar lipids (TPL) obtained from **A**: Olive pomace (OP), **B**: Fish oil diet (FO diet), and **C**: Olive pomace diet (OP diet) on NH_2_ column with UV detection at 208 nm, a flow rate of 3 mL min^−1^ and the elution solvent system 1 (see Experimental section).

The TPL of each of the five samples were diluted in chloroform/methanol (1:1) and fractionated by normal-phase HPLC in three sequential separations of 100 μL. The fractions with the same retention times from each injection were unified and examined for their biological activity to modulate PAF-induced washed rabbit platelet aggregation. The normal—phase HPLC chromatographs of polar lipid fractions of OP, FO diet, and OP diet are shown in Figure 1.

The aggregatory biological activities of the most potent polar lipid fractions of OP, FO diet, and OP diet were measured and expressed in peq PAF g^−1^ (Table 3).

**Table 3:** Biological activity of the most potent polar lipid fractions on washed rabbit platelets after normal-phase HPLC separation of olive pomace (OP), fish oil diet (FO diet), and olive pomace diet (OP diet) expressed as peq PAF g^−1^.

HPLC lipid fractions	OP	FO diet	OP diet
5	1.01 ± 0.05	0.04 ± 0.01 ^†^	1.24 ± 0.07 ^†^
6	0.43 ± 0.02	n.a.	0.56 ± 0.02
7	2.34 ± 0.12	n.a.	n.a.

Values are means of three individual measurements; results are expressed as mean ± SD (95% confidence limits); n.a.: No activity detected; ^†^: Indicates statistical significance within OF and OP diet, according to Wilcoxon test.

**Figure 2:**
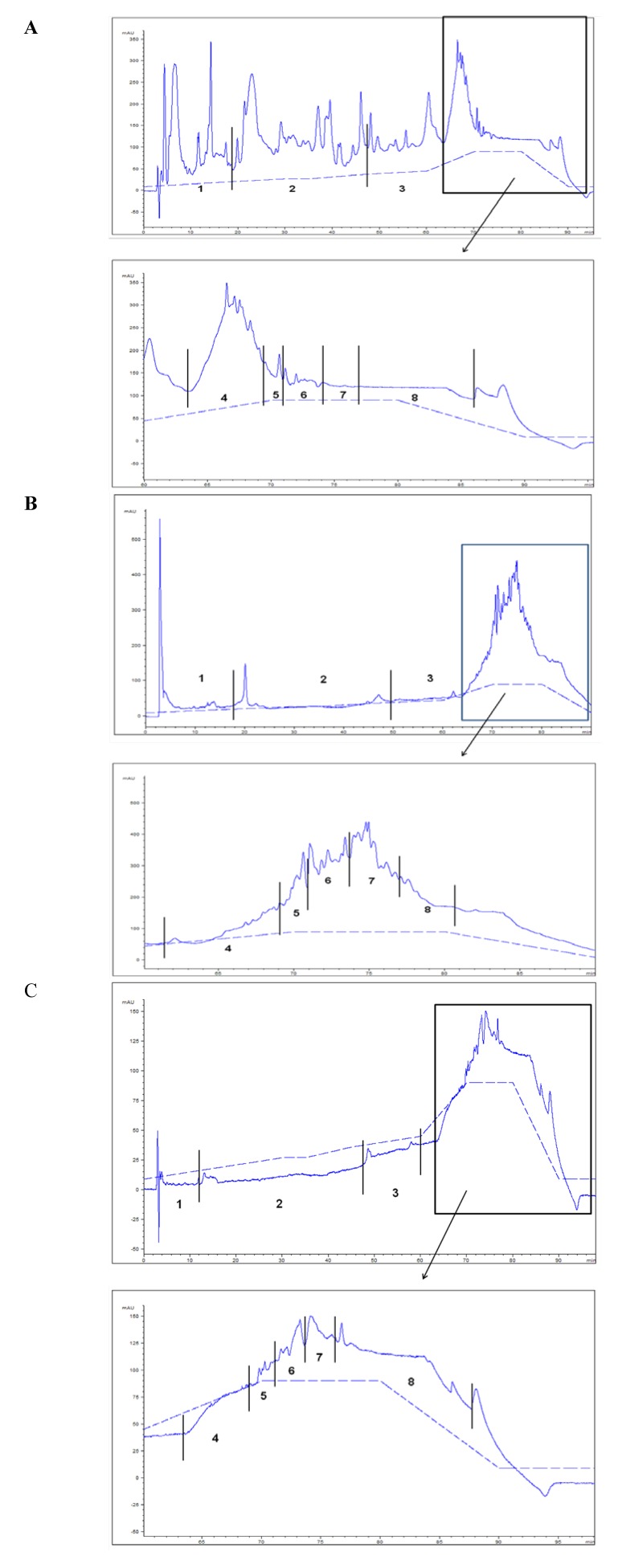
Representative chromatographs by reverse-phase HPLC separation of total polar lipids (TPL) obtained from **A**: Olive pomace (OP), **B**: fillets of aquacultured gilthead sea bream (*Sparus aurata*) fed with fish oil diet (FO diet), and **C**: fillets of aquacultured gilthead sea bream (*Sparus aurata*) fed with olive pomace diet (OP diet) on Nucleosil-300 C-18 column with UV detection at 208 nm, a flow rate of 1 mL min^−1^ and elution solvent system 2 (see Experimental section). Τhe last part of **Α**, **Β**, and **C** chromatograms has been expanded.

Concerning the polar lipid fractions obtained by normal-phase HPLC of aquacultured fish fed with FO diet, the most powerful activity was located in the fractions eluted from 120 to 140 min. Based on these data, these fractions were pooled together, evaporated under a nitrogen flow, diluted in chloroform/methanol (1:1), and further fractionated by reverse-phase HPLC with the above-mentioned solvent elution system 2 (see Experimental section). The individual fractions were tested for their PAF-like biological activity to induce platelet aggregation. The same process was followed for the polar lipid fractions of the aquacultured fish fed with OP diet, which also eluted from 120 to 140 min in the normal-phase HPLC separation. The reverse-phase HPLC chromatographs of polar lipid fractions of OP, aquacultured fish fed with FO and OP diets are shown in Figure 2.

The biological activities of the most active HPLC polar lipid fractions of fillets obtained from aquacultured gilthead sea bream fed with FO and OP diet (expressed as peq PAF g^−1^) are summarized in Table 4.

**Table 4:** Biological activity of polar lipid fractions on washed rabbit platelets after reverse-phase HPLC separation of fillets of aquacultured gilthead sea bream (*Sparus aurata)* fed with fish oil diet (FO diet) and olive pomace diet (OP diet) expressed as peq PAF g^−1^.

HPLC lipid fractions	Aquacultured fish fed with FO diet	Aquacultured fish fed with OP diet
1	0.13 ± 0.01 ^†^	0.30 ± 0.01 ^†^
2	n.a.	n.a.
3	0.04 ± 0.01	n.a.
4	0.11 ± 0.01 ^†^	0.25 ± 0.01 ^†^
5	0.11 ± 0.01 ^†^	0.24 ± 0.01 ^†^
6	n.a.	0.23 ± 0.01
7	0.11 ± 0.01 ^†^	0.26 ± 0.01 ^†^
8	0.16 ± 0.01 ^†^	0.28 ± 0.02 ^†^

Values are means of three individual measurements; results are expressed as mean ± SD (95% confidence limits); n.a.: No activity detected; ^†^: Indicates statistical significance within aquacultured fish fed with FO diet and aquacultured fish fed with OP diet, according to Wilcoxon test.

The data of Table 4, suggest a statistically significant increase of the biological activity of the polar lipid fractions present in aquacultured fish fed with OP diet when compared to the corresponding fractions of aquacultured fish fed with FO diet.

## 3. Discussion

There are plenty of studies demonstrating the effects of partial replacing of FO with vegetable oils [10,11], giving emphasis to the improvement of the altered fatty acid composition of fish muscle caused by vegetable oil in the diet, as well as the influence of the experimental fish diet on the quality and nutritional properties of the derived fish. According to recently published data [7,8], it is feasible to improve nutritional value and cardioprotective properties of gilthead sea bream by partially substituting FO in formulated fish feed with OP that has an increased phenolic content [12]; these phenols inhibit PAF-induced aggregation. Not only did the inclusion of OP in fish feed not decrease fish growth [7] it also improved fish lipid cardioprotective properties partly by increasing the activity of the PAF specific catabolism enzyme: PAF-specific acetylhydrolase (PAF-AH) [13].

According to Table 3 of our current study, polar lipid fractions 5 and 6 of OP diet had significantly increased biological activity against PAF-induced platelet aggregation when compared to the respective polar lipid fractions of FO diet with the same elution times. This result indicates that components of OP with *in vivo* antiatherogenic and cardioprotective qualities [14] maintained their strong cardioprotective activity while enriching the OP diet.

Moreover, it is worth mentioning that the HPLC polar lipid fraction 6 of aquacultured fish fed with OP diet (Table 4, Figure 2C)—which elutes in the area of phospholipids and glycolipids—caused a noticeable platelet aggregation. This result is in good agreement with some recent work of our group, where specific HPLC polar lipid fractions of sea bass (*Dicentrarchus labrax*) fed with OP diet—in which several phosphatidylcholine (PC) species have been structurally identified, that shows biological activity of fish lipids as agonists and/or antagonists of PAF-induced platelet activation [15]. Similarly other studies showed that polar lipid classes isolated from fish *Scomber scombrus* [16] and OP [14,17] have been found to exhibit analogous biological activities as agonists and/or antagonists of PAF-induced platelet activation. On the other hand, in the present work, the respective HPLC lipid fraction of aquacultured fish fed with FO diet with the same elution time (HPLC lipid fraction 6) did not display any biological activity (Table 4, Figure 2B).

Therefore, it could be suggested that the improved biological activity of the aforementioned HPLC polar lipid classes of aquacultured fish fed with OP diet could be attributed to the biologically active compounds present in OP enriched fish-feed and therefore in OP, that have elution times between 60–100 min (Figure 2A–C).

At this point, it should also be mentioned that these biological activities refer either to PAF-agonists or PAF-inhibitors which enhance and/or inhibit platelet aggregation caused by PAF. Natural PAF agonists are considered to be the best PAF inhibitors. These molecules act through PAF receptors, inhibiting PAF biological actions at low concentrations whilst inducing platelet aggregation at significantly higher concentrations (up to four orders of magnitude). However, these PAF-agonists are almost five orders of magnitude less potent than PAF in inducing PAF-like aggregation. These findings suggest that these compounds would minimize atherogenesis caused by PAF, by acting as PAF-inhibitors at the PAF receptors level in several cells and/or tissues [9,18].

The antiatherogenic properties of these agonists/inhibitors of either olive oil polar lipids or OP polar lipids were studied in cholesterol-fed rabbits where it was found that they not only significantly inhibited the development of atherosclerotic lesions, but also caused regression of the existing plaques, thus suggesting they are able to cure the existing atheromatosis [13,14,19].

## 4. Experimental Section

### 4.1. Reagents

All chemicals and reagents were of analytical grade purchased from Merck (Darmstadt, Germany) while bovine serum albumin (BSA) and PAF were obtained from Sigma (St Louis, MO, USA).

### 4.2. Samples

Five samples used for analysis: (a) OP—the solid by-product of the traditional olive oil extraction system, (b) FO diet containing FO as the predominant source of lipids, (c) OP diet where 8% of FO has been replaced by OP, and (d) aquacultured fish species *Sparus aurata* fed with the FO and OP diet. Both fish samples (fish fed with the FO and OP diet) obtained after the dietary experiment trial on gilthead sea bream conducted by Nasopoulou *et al.* [7].

The FO diet used was the same to the one used at the dietary experiment trial on gilthead sea bream conducted by Nasopoulou *et al.* [7], where the chemical composition of this diet was published.

OP originated from a local oil producer and the OP diet was formulated at the facilities of the marine farm where the dietary experiment took place. OP was added as dry material prior the extrusion. The pellets were dried, sealed and kept in air-tight bags until use.

### 4.3. Fish Diets’ Analysis

The reference diet (FO diet) contained 100% FO (cod liver oil) [7] while the experimental diet (OP diet) was formulated—following the principles of fish nutrition [20]—by substituting 8% of FO by OP. The chemical determinations of the OP diet were conducted according to EC 152/2009 Regulation [21], protein digestibility determination took place according to van Leeuwen *et al.* [22] and energy determination took place according to the following equation [20]: Energy (MJ/kg) = {(CPg × 23.6 kJ) + (CFg × 39.5 kJ) + ([CFig + NFEg] × 17.4 kJ)}/1000; where CP: Crude protein; CF: Crude fat; CFi: Crude fiber; NFE = 1000 − (CP + CF + Ash + Moist).

### 4.4. Instrumentation

HPLC separation was conducted on Total Polar Lipids (TPL) obtained from the samples, at room temperature, on an HP HPLC Series 1100 liquid chromatographer (Hewlett-Packard, Waidronnn, Germany) equipped with a 100 μL Loop Rheodyne (7725 i) loop valve injector, a degasser G1322A, a gradient pump G1311A, a HP UV spectrophotometer G1322A, as a detection system and a normal phase column YMC-Pack Amino, 250 × 20 mm, S-5 μm, 12 nm (internal diameter). For the purification of the most biologically active fractions obtained by normal-phase HPLC, a reverse-phase column Nucleosil-300 C-18 (250 × 4 mm, 7 μm) was used. The analysis of the chromatograph was performed via the Agilent Chemstation software. PAF-induced aggregation was measured in a Chrono-Log (Havertown, PA, USA) aggregometer coupled to a Chrono-Log recorder (Havertown, PA, USA).

### 4.5. Isolation of Lipids Extracts

Total lipids (TL) of OP, FO, and OP diet and fish fillets of aquacultured gilthead sea bream fed with FO and OP diet, were extracted by Bligh and Dyer method [23]. Their separation to Total Neutral Lipids (TNL) and TPL was achieved with the counter current distribution (CCD) extraction procedure [24]. The upper phase of petroleum ether contained the total neutral lipids while the lower phase of ethanol with total polar lipids were selected in a glass-stoppered flask, evaporated at 30 °C on the rotary evaporator, weighted, dissolved in chloroform/methanol (1:1), and stored at −20 °C until further analysis.

### 4.6. HPLC Separation of TPL

The separation of TPL from the samples was performed on a normal phase absorption NH_2_ column with a gradient elution system. Solvents and elution profile were the following (elution solvent system 1): Solvent A: Methanol (100%), solvent B: Acetonitrile (100%), and solvent C: Water (100%); elution profile: 0–55 min, 40:60 (A:B) (isocratic elution), followed by a linear gradient to 100% A from 55 to 60 min and maintained from 60 to 70 min, then a linear gradient to 100% C from 70 to 75 min and held from 75 to 105 min, followed by a linear gradient to 100% A from 105 to 110 min, then a linear gradient back to 40:60 (A:B) from 110 to 115 min and maintained at this ratio from 115 to 140 min. Injections of 50–100 μL of TPL of the samples were applied each time. The flow rate was 3 mL min^−1^ and the eluted substances were detected spectrophotometrically by UV detection at 208 nm at room temperature. In regards to the polar lipid fractions of OP, fish fed with FO diet and fish fed with OP diet, the strongest aggregatory activity was found at fractions with elution time from 120 to 140 min, which were pooled together and further separated on reverse-phase HPLC with Nucleosil-300 C-18 column to obtain potentially more highly purified polar lipid fractions. The separation was achieved on a stepped gradient elution system. Solvents and elution profile that were used are (elution solvent system 2): Solvent A: Methanol: acetic acid (90:1, v/v), solvent B: Acetonitrile (100%) and solvent C: Water: acetic acid (100:1, v/v); elution profile: 0–30 min, from 9:1:90 (A:B:C) to 27:3:70 (A:B:C) (gradient linear) and held from 30 to 35 min, followed by a linear gradient to 36:4:60 (A:B:C) over 35–45 min, then a linear gradient to 45:5:50 (A:B:C) from 45 to 60 min, followed by a linear gradient to 90:10 (A:B) from 60 to 70 min and held at this ratio from 70 to 80 min and then a linear gradient back down to 9:1:90 (A:B:C) from 80 to 90 min and maintained from 90 to 95 min [17]. Injections of 100 μL of polar lipid samples were applied each time. The flow rate was 1 mL min^−1^ and the eluted substances were detected spectrophotometrically by UV detection at 208 nm at room temperature.

### 4.7. Biological Assay

The purified polar lipid fractions of OP, FO, and OP diet and fish fillets of aquacultured fish fed with FO and OP diet obtained by the above HPLC separations, were tested for their biological activity according to the washed platelet aggregation assay [25]. PAF as well as the examined samples were dissolved in 2.5 mg of bovine serum albumin (BSA) mL^−1^ of saline. The biological activity of each lipid fraction to induce platelet aggregation was expressed in peq PAF g^−1^ in reference to an eight points’ regression curve of standard PAF [25].

### 4.8. Statistical Analysis

Chemical analyses were carried out in triplicates and all results were expressed as mean value ± SD. The Wilcoxon sign test was used to determine significant differences in the same group. Differences were considered significant for *p* < 0.05. The data were analyzed using a statistical software package (IBM SPSS Statistics 19.0, SPSS Inc., Chicago, IL, USA).

## 5. Conclusions

The data of the current study provide further biochemical evidence indicating that biologically active lipids of OP have effectively enriched both fish feed and aquacultured fish (*i.e.*, fish that has been fed with the aforementioned OP enriched fish feed). It could, thus, be suggested that OP inclusion in fish feed and the usage of this feed to produce aquacultured gilthead sea bream, has a dual beneficial impact: Firstly, OP enriches both fish feed and fish in polar lipids with cardioprotective properties and secondly, OP is valorized as a potential partial replacement of FO in the quest for sustainable production of fish feeds and increasing aquatic food security [6].

Conclusively, the present study shows that the use of OP for the partial replacement of FO as an alternative dietary lipid source in aquaculture fish feeds could increase the nutritional and subsequently the commercial value of fish feed and consequently those of aquacultured fish. Linking the data presented here to those on the cardioprotective properties of gilthead sea bream fed with OP diet [7,8], it could be suggested that the exploitation of OP in aquatic technology and the production of sustainable fish feeds is rather promising: while fish farming helps to meet growing demand for fish products worldwide, the use of OP in fish feeds has a positive environmental impact since a sustainable by-product of olive industry is valorised. Currently, we are focusing towards the structural elucidation of the cardioprotective fatty acids of marine origin and other lipid derivatives and the evaluation of the enriched fish feed and aquacultured fish in *in vivo* nutritional studies in animals and humans.

This work could also be useful in addressing satisfactorily the issue of aquatic food security, *i.e.*, providing sustainable and nutritious solutions for the aquaculture fish-feed sector with promising applications in both neutraceutical and pharmaceutical industries. Our current work is towards the creation of health claims—for these feeds and fish—against cardiovascular diseases following the corresponding guidelines of EFSA.

## Abbreviations

HPLC: High Performance Liquid Chromatography
EFSA: European Food Safety Authority
